# Design of Cocaethylene and Cocaine Conjugates to Produce Highly Selective Polyclonal Antibodies

**Published:** 2006-02

**Authors:** Caroline Gadjou, Yannic Danger, Pierre Sandouk, Jean-Michel Scherrmann, Dominique Blanchard, Gilles Folléa, Hervé Galons

**Affiliations:** 1 *Laboratoires de chimie organique et de Pharmacocinétique, 4, avenue de L’observatoire, Paris, France;*; 2 *Etablissement Français du Sang, Laboratoire de biotechnologie, 34 Boulevard Jean Monnet, Nantes;*; 3 *BioRun, 1 bis Boulevard des Américains, Nantes*

**Keywords:** cocaine, cocaethylene, benzoylecgonine, hapten, antibody

## Abstract

With the aim to obtain specific anti-cocaine antibodies directed against cocaine and active metabolites for use in immunotherapy, a series of six haptens were prepared, based on the structure of cocaine. The haptens differed by 3 positions of linkers: nitrogen, carboxyl group, and aromatic nucleus. The haptens were grafted onto 3 carrier proteins: bovine serum albumin, tetanus toxoid or keyhole limpet hemocyanin according to different methods of coupling: carbodiimide or mixed anhydride techniques. The immuno-conjugates were administered to rabbits and the antisera elicited were analyzed in term of titer, affinity and specificity. Variation in antisera properties were observed and attributed to the site of coupling the hapten, to the carrier proteins, and to the method of coupling. Antisera titers were in the range of 1/1 (no significant response) to 1/12,832, with antisera affinity up to 5.9 × 10^11^ M-1. This strategy allowed the selection of a new hapten, which after coupling on carrier proteins, led to the production of antisera with a high specificity toward cocaine and cocaethylene, but exclude the inactive metabolites of cocaine.

## INTRODUCTION

Cocaine is a powerfully addicting drug of abuse that has been increasingly associated with toxic consequences, a problem that is exacerbated by the lack of effective pharmacotherapies for treating cocaine overdose. Cocaine toxicity-related cardiac morbidity and mortality are due to several interacting mechanisms. Cocaine has a potent pharmacological effect, indirectly stimulating the sympathetic nervous system (1), and it has a direct toxic effect on the heart (2). Cardiovascular complications include myocardial ischemia and infarction (3, 4).

Toxic actions of cocaine are mediated through effects at multiple receptors (dopamine, norepinephrine and serotonin transporters) inducing a great obstacle for the classical receptor-antagonist approach. Up to date, they have contributed to the failure to devise specific treatments for cocaine overdose and addiction. This encouraged researchers to develop non-classical approaches including several immunological approaches for the treatment of cocaine overdoses (5). Catalytic antibodies have been able to reduce cocaine activity on heart (6). Alternatively, vaccination of animals with several cocaine-protein conjugate has induced a significant change in cocaine pharmacokinetics, inducing decreased levels of cocaine in the brain (7, 8). The crystal structure of complexes between monoclonal antibodies and cocaine has been reported (9, 10).

On a clinical point of view, cocaine abuse is in most cases associated with alcohol intake (11), resulting in an increase of the stimulative effects of cocaine (12). These stronger effects are due to the formation of cocaethylene a more active cocaine metabolite. Cocaethylene is also more toxic than cocaine: peak serum cocaethylene concentrations have been associated with prolonged myocardial depression (13). Toxicity on endothelial cells has been recently pointed out (14).

Cocaine is rapidly hydrolysed into inactive metabolites: benzoylecgonine, ecgonine methylester and finally ecgonine. In contrast to other metabolites, cocaethylene is more stable and its blood concentration is regularly found in higher concentration as compared to cocaine (15). The cardiotoxicity of cocaethylene has been studied in several animal models (16, 17).

**Figure 1:**
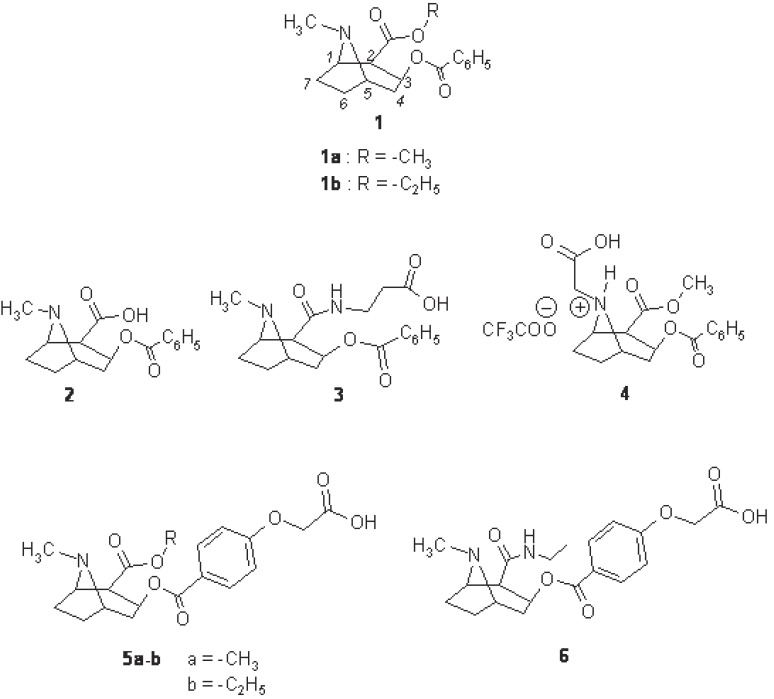
Structures of cocaine 1a, cocaethylene 1b, and synthetised haptens : benzoylecgonine 2, N - (benzoylecgonyl)-b-alanine 3, N - (acetic acid) -norcocaine trifluroacetate 4, 4’ - (oxyacetic acid) - benzoylecgonine methyl ester 5a, 4’ - (oxyacetic acid)-benzoylecgonine ethyl ester 5b, 4’ - (oxyacetic acid) - benzoylecgonine-N-ethylamide 6.

We embarked on a project to develop antibodies that could be used for an immunotherapy of cocaine overdoses. We report the selection of the antigen in order to develop antibodies directed against only cocaine and its toxic metabolites like cocaethylene. We present the study of the relation between hapten structure and cross-reactitivity of the antibodies produced by immunization of rabbits with hapten-carrier protein conjugates (Figure 1).

## MATERIALS AND METHODS

### Chemicals and instrumentation

Cocaine, 1a was purchased from Cooper (Melun, France). Benzoylecgonine, 2, and haptens 3-6 were prepared from cocaine by known methods (18, 19).

### Preparation of immunogens

The immunogens were prepared by coupling the six haptens to different carriers: bovine serum albumin (BSA: JRH Biosciences, Valbiotech, France; Mw=65,000 daltons), tetanus toxoid (TT: Pasteur-Mérieux Connaught, Marcy l’Etoile, France; MW=150,000 daltons), or keyhole limpet hemocyanin (KLH: Imject Mariculture Keyhole, Pierce, Interchim. KLH consist of several subunits of 450,000 daltons each, existing as a didecamer with an approximate molecular weight of 8 millions daltons. For each solution, protein titration was performed by the BCA protein assay (Pierce, Interchim).

The immunoconjugates were prepared using either the carbodiimide (EDC) (20), or the mixed anhydride (MA) techniques (21). The molar ratio of hapten/tetanus toxoid and hapten/BSA was 50:1, whereas the molar ratio for hapten/KLH was 100:1, based on 800,000 kDa for KLH. The hapten-carrier protein immunoconjugates were dialyzed overnight at +4°C in a dialysis cassette (Slide-A-Lyser cassette10K, Pierce, Interchim) against phosphate buffered saline, pH7.4, to remove unbound haptens, until no free drug was detectable by U.V. detection at 255 nm. The immunoconjugates were kept frozen at -20°C until use. The number of hapten molecules linked to the carrier protein (mole per mole) was determined by the trinitrobenzene sulfonic acid (TNBS) method (22).

### Production of antisera

Six groups of five New Zealand white female rabbits were immunized as previously described (23). Briefly, 0.5 mg of immunogens where injected subcutaneously into the back of each animal, in multiple points (5-8), with complete Freund adjuvant for the first administration, then with incomplete Freund adjuvant for the subsequent injections. Blood was withdrawn from marginal ear vein one week after each injection. Plasma were separated by centrifugation (4000 rpm/min, at +4°C), and antisera were analyzed for titer, affinity and specificity.

### Analysis of antisera

The titration of anti-cocaine antibodies was performed using a radio-immunoassay assay procedure. Serially diluted antisera were analyzed by competitive immunoassay using a determined quantity of 3H-cocaine (28,000 dpm, 0.96 pmol/tube) (levo-[benzoyl-3,4-3H(N)]-cocaine, ref: NET-510, 925GBq-1.85TBq/mmol, NEN, France). The separation of bound and free ligand from antigen-antibody complexes was performed by ammonium sulfate precipitation at half-saturation as previously described (24). The activity of the supernatant was measured by liquid scintillation counting (Tri-Carb liquid scintillation analyzer (Packard, France). Results were expressed as percentage of bound cocaine (B/AT-NS) versus antisera dilutions. The curve was fitted by using Graph-Pad Prism program (ISI, USA). The titers were defined as the inverse of the dilution of anti-cocaine antisera which gives 50% of [3H]-cocaine binding.

The affinity of the antisera was calculated according to the method of Müller (25).

The specificity of antibodies was determined by testing the cross-reactivity of cocaine or cocaine metabolites (cocaethylene, benzoylecgonine all products from Sigma) according to the RIA titer procedure described above, using concentrations in the range of 4,000 nmol/L to 0.002 nmol/L for cocaine and cocaine metabolites.

The cross-reactivity was expressed as a percentage ratio of cocaine concentration to the cross-reacting cocaine metabolites concentration at 50% inhibition of maximum binding.

## RESULTS

The analytical properties of the antisera in term of titer, affinity and specificity according to the different haptens are gathered in Table 1. The described values were obtained 4 to 6 months after priming the rabbits. Groups of 5-6 rabbits were tested for each hapten. Values are presented as a range. A large variation in titers was observed in each group of rabbits. But the maximal values of titer were obtained when rabbits were immunized with the 5a hapten linked to BSA by the mixed anhydride method (titer=7790-12832). The affinity constant (Ka) reached high values, up to 1010 - 1011 M-1, with 5a, 5b or 6 haptens linked either with BSA, KLH, or TT carrier protein, used in combination. Taking into account the titer and the affinity results, the mixed anhydride coupling method proved to be the most efficient method.

**Table 1:** Analytical properties of the rabbit polyclonal antibodies

Hapten	Protein carrier	Coupling method	Titer (RIA)	Affinity (M^-1^)	Specificity (%)	
					Benzoyl-ecgonine	Cocaethylene

2	BSA	EDC	25-669	3.5-5.0 × 10^8^	>100	96
3	BSA	EDC	318-450	0.8-1.6 × 10^9^	82-90	>100
	BSA	MA	221-2682	1.5-9.7 × 10^8^	0.3-10	35-59
	KLH	EDC	95-325	5.2-6.9 × 10^9^	40-54	>100
	KLH	MA	631-1653	1.5-3.8 × 10^9^	1-13	23-31
4	BSA	EDC	No	significant	response	
	KLH	EDC				
5a	BSA	MA	7790-12832	1-1.6 × 10^9^	45-62	57-58
	KLH	MA	79-198	6.2-9.2 × 10^9^	100	67-100
5b	BSA	MA	755-3382	1.5-5.9 × 10^11^	2.6-17	71-100
	KLH	MA	165-1408	1.4-1.7 × 10^9^	0.8-3	55-147
	TT	MA	277-940	1.2-5.3 × 10^10^	18-23	95-99
6	BSA	MA	508-1891	2.2-8.5 × 10^10^	13-16	12-75
	TT	MA	24-36	0.3-2.3 × 10^11^	0.8-1.2	18-86

BSA, Bovine Serum Albumin; KLH, Keyhole Limpet Hemocyanin; TT, Tetanus Toxoid; EDC, 1-Ethyl-3-[3-dimethylaminopropyl]-carbodiimide hydrochloride; MA, mixed anhydride.

For all antisera, the cross-reactivity of ecgonine-methyl ester was always <0.06% and <0.03% for ecgonine. In contrast, the cross-reactivity of benzoylecgonine, and cocaethylene, strongly depends on the nature of the hapten: coupling via the benzoyl group of the hapten 5b gave the most interesting cross-reactivity pattern in the aim of the immunoneutralization challenge, i.e., antisera with a low cross-reactivity with benzoylecgonine, and a high cross-reactivity with the toxic metabolite: cocaethylene.

## DISCUSSIONS

**Figure 2:**
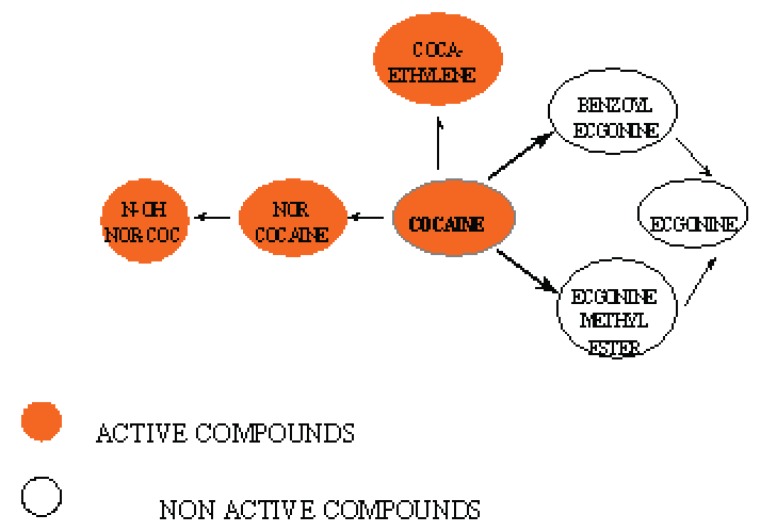
Main metabolites of cocaine. N-OH NOR-COC : N-hydroxynorcocaine.

As a consequence of the extensive metabolism of cocaine into toxic and non toxic compounds, the specificity of the therapeutic antibodies has to be oriented towards the recognition of the toxic compounds only, i.e. cocaine and cocaethylene being formed following cocaine and alcohol consumptions (Figure 2). More than 80 % of cocaine are biotransformed to non toxic compounds (benzoylecgonine, ecgonine methyl ester and ecgonine) which should not be recognised by the “ideal” therapeutic anti-cocaine antibody.

Based upon this immuno neutralization pattern only one group of haptens (5) achieved to only bind cocaine and cocaethylene. The other types of haptens did not allowed to obtain antibodies for therapeutic use. To the best of our knowledge, production of antibodies directed against cocaethylene has not been disclosed. As yet evoked, the most selective antibodies were obtained starting from hapten 5b. Two explanations can be found to explain the efficiency of this hapten. At first, the position of the linkage which allows the recognition of the ethyl ester group explains the low cross reactivity with benzoylecgonine of the antibodies obtained from 5b. Secondly, due to the presence of two esters groups which activates each over, cocaine is known to display only little stability in water even at neutral pH. In the case of haptens 5, the electrodonating oxygen limits the reactivity of the ester and contributes to the stability of the immunogen.

Numerous antigens have been prepared from cocaine for the development of immunoassays. In most cases, haptens are derived from benzoylecgonine (26). Conjugates linked on the tropane nitrogen have also been used (27). The cross reactivities of the antibodies are usually not disclosed. A 30 % cross-reactivity with benzoylecgonine have been reported using a conjugate linked on the nitrogen (28). If the specificity properties were the first criterion of hapten selection, the affinity must be also considered as a critical element for successfully development of antibodies to be used for immuno toxicotherapy of cocaine. It has been clearly demonstrated that affinity constants above 10^9^ M^-1^ are leading to stable drug-antibody complex allowing an efficient immuno-neutralization of other haptens like digoxin, colchicine and tricyclic antidepressants (29, 30).

In conclusion, new immunoconjugates of cocaine was prepared with the goal to develop antibodies able to bind both cocaine and cocaethylene. Conjugates derived from hapten 5b led to antibodies that fulfilled requirements. They also allowed us to obtain highly selective monoclonal antibodies (31). These antibodies could represent powerful new tools for therapeutic use in immunotherapy of cocaine overdoses.

## References

[1] Bunney EB, Appel SB, Brodie MS (2001). Electrophysiological effects of cocaethylene, cocaine, and ethanol on dopaminergic neurons of the ventral tegmental area. J. Pharmacol. Exp. Ther.

[2] Zhang L, Xiao Y, He J (1999). Cocaine and apoptosis in myocardial cells. Anat. Rec.

[3] Benzaquen BS, Cohen V, Eisenberg MJ (2001). Effects of cocaine on the coronary arteries. Am. Heart J.

[4] Roldan CA, Aliabadi D, Crawford MH (2001). Prevalence of heart disease in asymptomatic chronic cocaine users. Cardiology.

[5] Kosten T, Owens SM (2005). Immunotherapy for the treatment of drug abuse. Pharmacol. Ther.

[6] Briscoe RJ, Jeanville PM, Cabrera C, Baird TJ (2001). A catalytic antibody against cocaine attenuates cocaine’s cardiovascular effects in mice: a dose and time course analysis. Int. Immunopharmacol.

[7] Kantak KM, Collins SL, Lipman EG, Bond J (2000). Evaluation of anti-cocaine antibodies and a cocaine vaccine in a rat self-administration model. Psychopharmacology (Berl).

[8] Carrera MRA, Trigo JM, Wirsching P, Roberts AJ (2005). Pharmacol. Biochem. Behav.

[9] Larsen NA, Zhou B, Heine A, Wirschung P (2001). Crystal structure of a cocaine-binding antibody. J. Mol. Biol.

[10] Pozharski E, Moulin A, Hewagama A, Shanafelt AB (2005). Diversity in hapten recognition: structural study of an anti-cocaine antibody M82G2. J. Mol. Biol.

[11] Pennings EJ, Leccese AP, Wolff FA (2002). Effects of concurrent use of alcohol and cocaine. Addiction.

[12] Raven MA, Necessary BD, Danluck DA, Ettenberg A (2000). Comparison of the reinforcing and anxiogenic effects of intravenous cocaine and cocaethylene. Exp. Clin. Psychopharmacol.

[13] Kalasinsky KS, Bosy TZ, Schmunk GA, Ang L (2000). Regional distribution of cocaine in postmortem brain of chronic human cocaine users. J. Forensic. Sci.

[14] Tacker DH, Okorodudu AO (2004). Evidence for injurious effect of cocaethylene in human microvascular endothelial cells. Clin. Chim. Acta.

[15] Blaho K, Logan B, Winbery S, Park L (2000). Blood cocaine and metabolite concentrations, clinical findings, and outcome of patients presenting to an ED. Am. J. Emerg. Med.

[16] Wilson LD, Henning RJ, Suttheimer C, Lavins E (1995). Cocaethylene causes dose-dependent reductions in cardiac function in anesthetized dogs. J. Cardiovasc. Pharmacol.

[17] O’Leary ME (2002). Inhibition of HERG potassium channels by cocaethylene: a metabolite of cocaine and ethanol. Cardiovasc. Res.

[18] Martinet F, Pham Huy C, Galons H, Tomas A (1997). Regioselective hydrolysis of cocaine, convenient acylation procedure by benzoylecgonine. Synthetic. Commun.

[19] Schermann JM, Pouletty P, Galons H (2003). Cocaethylene immunogens and antibodies. US. Patent.

[20] Sheenan JC, Hess GP (1955). A new method for forming peptide bonds. J. Amer. Chem. Soc.

[21] Erlanger BF, Borek F, Beiser SM, Lieberman S (1957). Steroid-protein conjugates, Preparation and characterization of conjugates of bovine serum albumin with testosterone and with cortisone. J. Biol. Chem.

[22] Snyder SL, Sobocinski PZ (1975). An improved 2,4,6-trinitrobenzenesulfonic acid method for the determination of amines. Anal. Biochem.

[23] Hurn BAL, Chantler SM, Helen Van Vunakis, John J (1980). Production of reagent antibodies. Methods in Enzymology, Immunochemical techniques part A.

[24] Chard T, Helen Van Vunakis, John J (1980). Ammonium sulfate and P.E.G as reagents to separate antigen from antigen-antibody complexes. Methods in Enzymology, vol 70, Immunochemical techniques part A.

[25] Muller R (1980). Calculation of average antibody affinity in anti-hapten sera from data obtained by competitive radioimmunoassay. J. Immunol. Methods.

[26] Matsushita M, Hoffman TZ, Ashley JA, Zhou B (2001). Cocaine catalytic antibodies: the primary importance of linker effects. Bioorg. Med. Chem. Lett.

[27] Koetzner L, Deng S, Sumpter TL, Weisslitz M (2001). Titer-dependent antagonism of cocaine following active immunization in rhesus monkeys. J. Pharmacol. Exp. Ther.

[28] Yugawa K, Sigetoh N, Miazaki J, Mitsumata T (2001). US. Patent.

[29] Chappey O, Niel E, Debray M, Wautier JL (1995). Efflux of intra-cellular colchicines in lymphocytes with colchicines-specific Fab fragments. J. Pharmacol. Exp. Ther.

[30] Cano NJ, Sabouraud AE, Benmoussa K, Roquet F (1995). Monoclonal digoxin-specific antibodies induce dose- and affinity-dependent plasma digoxin redistribution in rats. Pharm. Res.

[31] Danger Y, Devys A, Gadjou C, Galons H (2004). Developement of monoclonal antibodies directed against cocaine and cocaethylene : Potential new tools for immunotherapy. Hybridoma. and hybridomics.

